# Higher-order p-Laplacian boundary value problem at resonance on an unbounded domain

**DOI:** 10.1016/j.heliyon.2020.e04826

**Published:** 2020-09-22

**Authors:** Samuel A. Iyase, Kanayo S. Eke

**Affiliations:** Department of Mathematics, Covenant University, Canaanland, KM 10 Idiroko Road, P. M. B. 1023, Ota, Ogun State, Nigeria

**Keywords:** Mathematics, Higher-order, Resonance, p-Laplacian, Unbounded domain, Coincidence degree

## Abstract

In this work, we employ the extension of Mawhin's coincidence degree by Ge and Ren to investigate the solvability of the p-Laplacian higher-order boundary value problems of the form

$\begin{array}{l} {\left(w\left(t\right)\phi_{p}\left(x^{\left(n-1\right)}\left(t\right)\right)\right)}^{'}=h\left(t,\;x\left(t\right),\cdots ,\;x^{\left(n-1\right)}\left(t\right)\right),\;0<t<\infty ,\\ x^{\left(n-2\right)}\left(0\right)=\frac{\left(n-2\right)!}{\eta^{n-2}}x\left(\eta \right),\;x^{\left(n-1\right)}\left(0\right)=x^{\left(i\right)}\left(0\right)=0,\;i=1,2,\;\cdots ,\;n-3,\;n\geq 3, \end{array}$

$x^{\left(n-2\right)}\left(\infty \right)=\int_{0}^{\eta }x^{\left(n-2\right)}\left(s\right)dA\left(s\right),$

Where $\eta >0,\;h:\left[0,\;\infty \right)\;\times {\mathbb{R}}^{n}\to \mathbb{R}$ is a Caratheodory's function with A(0)=0, A(η)=1, w ∈C[0, ∞), w(t)>0 for all $t\;\text{ ≥ }0,{\text{ ϕ}}_{p}\left(s\right)={\left|s\right|}^{p-2}s$.

## 1 Introduction

In this paper we obtain existence results for the following higher-order p-Laplacian boundary value problems

(1.1) $${\left(w\left(t\right)\phi_{p}\left(x^{\left(n-1\right)}\left(t\right)\right)\right)}^{'}=h\left(t,\;x\left(t\right),\cdots ,\;x^{\left(n-1\right)}\left(t\right)\right),\;0<t<\infty ,$$

subject to the following boundary conditions

(1.2) $$\begin{array}{l} x^{\left(n-2\right)}\left(0\right)=\frac{\left(n-2\right)!}{\eta^{n-2}}x\left(\eta \right),\;x^{\left(n-1\right)}\left(0\right)=x^{\left(i\right)}\left(0\right)=0,\;i=1,2,\;\cdots ,\;n-3,\;n\geq 3\;,\;\\ x^{\left(n-2\right)}\left(\infty \right)=\int_{0}^{\eta }x^{\left(n-2\right)}\left(s\right)dA\left(s\right). \end{array}$$

where $\eta >0,\;h:\left[0,\;\infty \right)\;\times {\mathbb{R}}^{n}\to \mathbb{R}$ is a Caratheodory's function with respect to $L^{1}\left[0,\;\infty \right).\;A:\left[0,\;\eta \right]\to \left[0,\infty \right)$ is a non-decreasing function with A(0)=0, A(η)=1, w∈C[0,∞) with w(t)>0 for all $t\geq 0,\;\phi_{p}\left(s\right)={\left|s\right|}^{p-2}s,\;p>1,\phi_{p}^{-1}=\phi_{q},\;q>1$.

The study of boundary value problems with a p-Laplacian is important because they have applications such as in non-Newtonian mechanics, nonlinear elasticity, bloodflow models, glaciology etc. Higher-order boundary value problems at resonance when p = 2 have received much attention in the literature e.g [2, 4, 5, 7, 10]. Most of the results were obtained using Mawhin's coincidence degree arguments [11]. However when p≠2, the differential operator is no longer linear and Mawhin's continuation theorem can no longer be applied directly. On the other hand, higher-order p-Laplacian boundary value problems on unbounded domains at resonance have not received much attention in the literature. Most of the studies in this direction have concentrated mainly on bounded domains. For some recent results on boundary value problems with a p-Laplacian, see [6, 8, 9, 12, 13, 15, 16] and the references therein.

The boundary value problem 1.1), 1.2) is a problem at resonance if $\;Lx={\left(w\left(t\right)\phi_{p}\left(x^{\left(n-1\right)}\left(t\right)\right)\right)}^{'}=0$ has nontrivial solutions under the boundary conditions (1.2). If Lu=0 has only the trivial solutions then 1.1), 1.2) is then said to be at nonresonance. In general, resonance problems can be written in the abstract form Lx=Nx where L is not invertible. This paper is organized as follows. In section 2 we recall some preliminary definitions, theorems and Lemmas. Section 3 will be devoted to stating and proving the main existence results and in section 4 we provide an example to validate our result.

## 2 Preliminaries

In this section we introduce the theoretical foundations of the methods we shall utilize in the subsequence sections.

**Definition 2.1:** Let X and Z be Banach spaces with norms $||\cdot |{|}_{X},\;||\cdot |{|}_{Z}$ respectively. An operator L:X∩dom L→Z is said to be quasi-linear if

(i) ImL=L(X∩domL) is a closed subset of Z,
(ii) kerL={x∈X∩domL:Lx=0} is linearly homeomorphic to ${\mathbb{R}}^{n}$.

**Definition 2.2:** Let X be a Banach space and $X_{1}\subset X$ a subspace. A mapping $Q:\;X\;\to X_{1}$ is called a semi-projector if

(i) $Q^{2}x=Qx,\;x\in X$,
(ii) Q(λx)=λ Qx, x∈X, λ∈ℝ.

**Definition 2.3:** $N_{\lambda }:\bar{\Omega }\to Z,\;\lambda \in \left[0,\;1\right]$ is said to be L-compact in $\bar{\Omega }$ if there exists $Z_{1}\subset \;Z$ with $dimZ_{1}=\;dim\;ker\;L$ and the operators Q and R such that the following conditions hold

(2.1) (a)ker Q = ImL,

(2.2) $$\left(\text{b}\right)\;QN_{\lambda }x=0,\;\lambda \in \;\left(0,1\right)\text{, if and only if}\;QNx=0$$

(2.3) $$\left(c\right)\;R\left(.,0\right)\;is\;the\;zero\;operator,$$

(2.4) $$\left(\text{d}\right)\;R\left(.\;,\;\lambda \right){|}_{\Sigma_{\lambda }}=\left(1\;-P\right){|}_{\Sigma \lambda },$$

Where $\;\Sigma_{\lambda }=\left\{x\in \bar{\Omega }\;:\;Lx=N_{\lambda }\;x\right\}$,

(2.5) $$\left(\text{e}\right)\;L\left[P+R\left(.\;,\lambda \right)\right]=\left(1-Q\right)N_{\lambda },$$

where P:X→X is a projector and Q is a semi-projector such that ImP= ker L and

$ImQ\;=\;Z_{1}$.

We shall use the following inequalities [14] in the context of the p-Laplacian $\phi_{p}\left(s\right),\;p>1$,

(2.6) $$\left(\text{i}\right)\;\phi_{p}\left(u\;+\;v\right)\leq \phi_{p}\left(u\right)\;+\phi_{p}\left(v\right),\;if\;1<p\leq 2.$$

(2.7) $$\left(\text{ii}\right)\;\phi_{p}\left(u\;+\;v\right)\leq \;2^{p-2}\left(\phi_{p}\left(u\right)\;+\phi_{p}\left(v\right)\right),\;if\;p>\;2.$$

**Definition 2.4:** The map $h:\left[0,\;\infty \right)\;\times {\mathbb{R}}^{n}\to \mathbb{R}$ is $L^{1}$- Caratheodory if the following conditions hold,

(i) for each $\;x\in {\mathbb{R}}^{n}\;$, the mapping t→h(t, x) is Lebesgue measurable,
(ii) for a.e. t∈ [0,∞), the mapping x→h(t, x) is continuous on ${\mathbb{R}}^{n}$,
(iii) for each r > 0, there exists a $\psi_{r}\in \;L^{1}\left[0,\infty \right)$ such that for a.e. t∈ [0,∞) and every x such that |x|≤r  we have $\left|h\left(t,\;x\right)\right|\leq \psi_{r}\left(\text{t}\right)$.

In what follows we shall use the following result of Ge and Ren.

**Theorem 2.1** [6]**:** Let X and Z be Banach spaces with norms $||\cdot |{|}_{X},||\cdot |{|}_{Z}$ and V⊂X be an open and bounded non empty set. Suppose L:X ∩dom L→Z is a quasi-linear operator and $N_{\lambda }:\bar{V}\to Z,\lambda \in \;\left[0,\;1\right]$ is L-compact. In addition, if the following conditions are satisfied;

(1) $Lx\;\neq N_{\lambda }x,\;x\in \partial V\cap \;dom\;L,\;\lambda \in \left(0,\;1\right),$
(2) deg (JQN, V∩ker L, 0)≠0,

where $N=N_{1}$ and J:ImQ →ker L is a homeomorphism with J(0)=0 and deg is the Brouwer degree. Then the abstract equation Lx =Nx has at least one solution in $dom\;L\cap \bar{V}\;$.

Let $D_{\infty }\;=\;\{x:\;\left[0,\;\infty \right)\;\to \mathbb{R}$ x is continuous on [0, ∞) and $\;lim_{t\to \infty }x\left(t\right)$ exists}. For $x\in \;D_{\infty }$ define $||x|{|}_{\infty }=\;Sup_{t\in [0,\infty )}\left|x\left(t\right)\right|$.

Then $D_{\infty }$ is a Banach space (see [1, 3]).

**Lemma 2.1:** Let $X=\{x:\;\left[0,\;\infty \right)\;\to \mathbb{R}:\;x,\text{ w}\phi_{p}\left(x^{\left(n-1\right)}\right)\in A\;C\;\left[0,\infty \right)$, $\;lim_{t\to \infty }e^{-t}\left|x^{\left(i\right)}\left(t\right)\right|$ exists,

(2.8) $$\;0\leq i\leq n-1,\;{\left(\text{w}\left(t\right)\phi_{p}\;\left(x^{\left(n-1\right)}\left(t\right)\right)\right)}^{'}\in \;L^{1}\left[0,\infty \right)\},$$

With norm,

(2.9) $$\left|\right|\text{x}\left|\right|=\max_{0\leq i\leq n-1}\left(sup_{t\in \;[0,\;\infty )}\;e^{-t}\left|x^{\left(i\right)}\left(t\right)\right|\right).$$

Then X is a Banach space.

**Proof:** First we show that ||⋅|| defines a norm. Clearly ||x||>0 and ||x||=0 if and only if $(sup_{t\in [0,\infty )}\;e^{-t}\left|x^{\left(i\right)}\left(t\right)\right|=0$ if and only if $x^{\left(i\right)}=0,\;i=0,1,2,3,\cdots ,\;n-1\;$.Let λ∈ℝ. Then,

$$\begin{array}{ll} \left|\right|\lambda x\left|\right| & =\max_{0\leq i\leq n-1}(sup_{t\in [0,\infty )}e^{-t}\left|{\left(\lambda x\right)}^{\left(i\right)}\left(t\right)\right|)\\ & =\left|\;\lambda \right|\max_{0\leq i\leq n-1}(sup_{t\in [0,\infty )}e^{-t}\left|x^{\left(i\right)}\left(t\right)\right|)\\ & =\left|\;\lambda \right|\left||x|\right|. \end{array}$$

$$\begin{array}{ll} \left|\right|x+y\left|\right| & =\max_{0\leq i\leq n-1}\left(sup_{t\in [0,\infty )}\;e^{-t}\left|{\left(x\left(t\right)\;+\;y\left(t\right)\right)}^{\left(i\right)}\right|\right)\\ & =\;\max_{0\leq i\leq n-1}\left(sup_{t\in [0,\infty )}\;e^{-t}\left|\left(x^{\left(i\right)}\left(t\right)\;+\;y^{\left(i\right)}\left(t\right)\right)\right|\right)\\ & \leq \;\max_{0\leq i\leq n-1}\left(sup_{t\in [0,\infty )}\;e^{-t}\left|x^{\left(i\right)}\left(t\right)\;\right|\right)+\max_{0\leq i\leq n-1}\left(sup_{t\in [0,\infty )}\;e^{-t}\left|y^{\left(i\right)}\left(t\right)\right|\right)\\ & \leq \left|\right|x\left|\right|+\left|\right|y\left|\right|. \end{array}$$

Next we prove that X is complete.

Let $\left\{x_{k}\right\}$ be a Cauchy sequence in X. Then $\left\{e^{-t}x_{k}\right\}$ and $\left\{e^{-t}x{'}_{k}\right\}$ are Cauchy sequences in $D_{\infty }$. Thus there exists $y_{0}^{*}$ and $y_{1}^{*}$ in $D_{\infty }$ such that

$$lim_{k\to \infty }\;sup_{t\in [0,\infty )}\left|\;e^{-t}x_{k}\;-\;y_{0}^{*}\right|\;=\;lim_{k\to \infty }\;sup_{t\in [0,\infty )}\;\left|e^{-t}x{'}_{k}-\;y_{1}^{*}\right|=0.$$

Let $\;x_{0}^{*}\left(t\right)=e^{t}y_{0}^{*}$ then

$$\;lim_{k\to \infty }\;sup_{t\in [0,\infty )}\;e^{-t}\left|x_{k}(t)-x_{0}^{*}(t)\right|=0.$$

Set $x_{1}^{*}\left(t\right)=e^{t}y_{1}^{*}\left(t\right)\;$ then $\;lim_{k\to \infty }sup_{t\in [0,\infty )}\;e^{-t}\left|x_{k}^{'}\left(t\right)\;-\;x_{1}^{*}\left(t\right)\right|=0$.

Since the convergence to $\;\;x_{0}^{*}\left(t\right)$ and $\;\;x_{1}^{*}\left(t\right)$ is uniform on [0, M] for any M>0, then $\;\;x_{0}^{*}\left(t\right)$ is differentiable on [0, M] and $\;\left(\;x_{0}^{*}\right)'\;=\;x_{1}^{*}$. Since M is arbitrary $\;x_{0}^{*}$ is differentiable on [0, ∞). Following the same reasoning we obtain

$$x_{i}^{*}=\;{\left(\;x_{0}^{*}\right)}^{\left(i\right)},\;i=0,\;1,\;2,\;\cdots ,\;n-1\text{ .}$$

Hence,

$$lim_{k\to \infty }\;sup_{t\in [0,\infty )}\;e^{-t}\left|x_{k}^{\left(i\right)}-\;x_{i}^{*}\right|=0.$$

Now

$$\begin{array}{ll} lim_{k\to \infty }\left||x_{k}-\;x_{0}^{*}|\right| & =lim_{k\to \infty }\max_{0\leq i\leq n-1}\;\left(sup_{t\in [0,\infty )}\;e^{-t}\left|{\left(x_{k}-\;x_{0}^{*}\right)}^{\left(i\right)}\right|\right)\\ & =lim_{k\to \infty }\max_{0\leq i\leq n-1}\;\left(sup_{t\in [0,\infty )}\;e^{-t}\left|{\left(x_{k}\right)}^{\left(i\right)}-\;{\left(x_{i}^{*}\right)}^{\left(i\right)}\right|\right)=0. \end{array}$$

Also

$$w\left(t\right)\phi_{p}\left({\left(\;x_{0}^{*}\left(t\right)\right)}^{\left(n-1\right)}\right)=w\left(t\right)\phi_{p}{\left(e^{t}y_{0}^{*}\right)}^{\left(n-1\right)}\in \;A\;C\;\left[0,\;\infty \right),$$

and

$$w\left(t\right)\phi_{p}\left({\left(\;x_{0}^{*}\right)}^{\left(n-1\right)}\right)'=\left(w\left(t\right)\phi_{p}{\left(e^{t}y_{0}^{*}\right)}^{\left(n-1\right)}\right)'\;\in L^{1}\left[0,\infty \right).$$

Thus $x_{0}^{*}\in X\;$ and hence X is a Banach space.Let $Z=L^{1}\left[0,\infty \right)$ with the norm,

(2.10) $$||y|{|}_{1}=\int_{0}^{\infty }\;\left|y\left(t\right)\right|dt,\;y\;\in Z.$$

To derive the compactness of the operator R we use the following compactness criterion.

**Theorem 2.2** [2]**:** Let X be the space of all bounded continuous vector valued functions on [0,∞) and  V⊂X. Then V is relatively compact if the following conditions hold,

(i) V is bounded in X,
(ii) the functions from V are equicontinuous on any compact interval of [0,∞),
(iii) the functions from V are equiconvergent at infinity.

Let L: dom L ⊂ X→Z be defined by

(2.11) $$\;L\;x\left(t\right)=\;\left(\text{w}\left(t\right)\phi_{p}\left(x^{\left(n-1\right)}\left(t\right)\right)\right)',\;t\in \left[0,\infty \right),$$

Where

(2.12) $$\begin{array}{l} \;\\ domL=\left\{ \begin{array}{c} x\in X:x^{\left(n-2\right)}\left(0\right)=\frac{\left(n-2\right)!}{\eta^{n-2}}x\left(\eta \right),\\ x^{\left(n-1\right)}\left(0\right)=x^{\left(i\right)}\left(0\right)=0,\;i=1,2\cdots ,n-3,\\ x^{\left(n-2\right)}\left(\infty \right)=\int_{0}^{\eta }x^{\left(n-2\right)}\left(s\right)dA\left(s\right). \end{array}\right.\} \end{array}$$

We define $N_{\lambda }:X\;\to Z\;$ by

(2.13) $$N_{\lambda }x\left(t\right)=\lambda h\left(t,\;x\left(t\right),\;\cdots ,\;x^{\left(n-1\right)}\left(t\right)\right).$$

Then 1.1), 1.2) takes the abstract form,

(2.14) $$Lx\;=\;N_{\lambda }x\;\;\text{when}\;\;\lambda =\;1.$$

**Lemma 2.2:** If $x^{\left(n-2\right)}\left(\infty \right)=\int_{0}^{\eta }x^{\left(n-2\right)}\left(s\right)dA\left(s\right),\;x^{\left(n-2\right)}\left(0\right)=\frac{\left(n-2\right)!}{\eta^{n-2}}x\left(\eta \right)$,

$$x^{\left(n-1\right)}\left(0\right)=x^{\left(i\right)}\left(0\right)=0,\;i=1,2,\cdots ,\;n-3,$$

A(η)=1,  A(0)=0 then

(i) $ker\;L=\left\{x\;\in dom\;L:\;x\left(t\right)=ct^{n-2}\text{, }c\;\in R,\;t\in \left(0,\infty \right)\right\}$.
(ii) $ImL\;=\left\{y\in Z:\;\int_{0}^{\infty }\phi_{q}(\frac{1}{w\left(s\right)})\;\phi_{q}\left(\int_{0}^{s}y\left(\tau \right)d\tau \right)ds-\;\int_{0}^{\eta }\int_{0}^{t}\phi_{q}\left(\frac{1}{w(s)}\right)\;\phi_{q}\left(\int_{0}^{s}y(\tau )d\tau \right)dsdA(t)=0\right\}$.
(iii) L: domL→Z is a quasi-linear operator.

**Proof:** It is easily verified that $\;ker\;L=\left\{x\in domL:\;x\left(t\right)=\;ct^{n-2}\right\}$ hence (i) holds. To prove (ii), let  y∈Z and consider the equation,

(2.15) $$\left(w\left(t\right)\phi_{p}\left(x^{\left(n-1\right)}\left(t\right)\right)\right)'=y\left(t\right).$$

We prove that (2.15) has a solution x(t) that satisfies the boundary conditions (1.2) if and only if

$$\int_{0}^{\infty }\phi_{q}\left(\frac{1}{w\left(s\right)}\right)\phi_{q}\left(\int_{0}^{s}y\left(\tau \right)d\tau \right)ds-\;\int_{0}^{\eta }\int_{0}^{t}\;\phi_{q}\left(\frac{1}{w\left(s\right)}\right)\phi_{q}\left(\int_{0}^{s}y\left(\tau \right)d\tau \right)dsdA\left(t\right)=0.$$

From (2.15) we obtain

(2.16) $$x\left(t\right)=x\left(0\right)\;+\frac{1}{\left(n-2\right)!}\;\int_{0}^{t}{\left(t-s\right)}^{n-2}\phi_{q}\left(\frac{1}{w\left(s\right)}\right)\phi_{q}\left(\int_{0}^{s}y\left(\tau \right)d\tau \right)ds\;+\;ct^{n-2}.$$

Then,

$$x^{\left(n-2\right)}\left(t\right)=\int_{0}^{t}\phi_{q}\left(\frac{1}{\omega (s)}\right)\phi_{q}\left(\int_{0}^{s}y(\tau )d\tau \right)ds\;+\;x^{\left(n-2\right)}\left(0\right).$$

$$\begin{array}{ll} x^{\left(n-2\right)}\left(\infty \right) & =x^{\left(n-2\right)}\left(0\right)\;+\;\int_{0}^{\infty }\phi_{q}\left(\frac{1}{w\left(s\right)}\right)\phi_{q}\left(\int_{0}^{s}y\left(\tau \right)d\tau \right)ds\\ & =\int_{0}^{\eta }\left[x^{\left(n-2\right)}\left(0\right)\;+\;\int_{0}^{s}{\text{w}}_{q}\left(\frac{1}{\text{w}\left(v\right)}\right)\phi_{q}\left(\int_{0}^{v}y\left(\tau \right)d\tau \right)dv\right]dA\left(s\right)\\ & =x^{\left(n-2\right)}\left(0\right)A\left(\eta \right)\;+\;\int_{0}^{\eta }\int_{0}^{s}\phi_{q}\left(\frac{1}{w\left(v\right)}\right)\phi_{q}\left(\int_{0}^{v}y\left(\tau \right)d\tau \right)dv\;dA\left(s\right), \end{array}$$

and hence,

(2.17) $$\int_{0}^{\infty }w_{q}\left(\frac{1}{w\left(s\right)}\right)\phi_{q}\left(\int_{0}^{s}y\left(\tau \right)d\tau \right)ds\;-\;\int_{0}^{\eta }\int_{0}^{t}\phi_{q}\left(\frac{1}{w\left(s\right)}\right)\phi_{q}\left(\int_{0}^{s}y\left(\tau \right)d\tau \right)dv\;dA\left(t\right)=0.$$

If (2.17) holds then,

(2.18) $$x\left(t\right)=\;ct^{n-2}\;+\;\frac{1}{\left(n-2\right)!}\int_{0}^{t}{\left(t-s\right)}^{n-2}\phi_{q}\left(\frac{1}{w\left(s\right)}\right)\phi_{q}\left(\int_{0}^{s}y\left(\tau \right)d\tau \right)ds.$$

where c an arbitrary constant is the solution of (2.15). Thus  dim ker L=1<∞, ImL⊂ Z is closed. Hence L is a quasi-linear operator.

**Lemma 2.3:** If h is a $\;L^{1}$-Caratheodory's function then $N_{\lambda }:\;\varpi \to Z$ is L-compact in ϖ, ω⊂X an open and bounded subset with the origin 0∈ω.

**Proof:** Let Q: Z→Z be defined by

(2.19) $$Qy\left(t\right)=\rho \left[\int_{0}^{\infty }\phi_{q}\left(\frac{1}{w\left(s\right)}\right)w_{q}\left(\int_{0}^{s}y\left(\tau \right)d\tau \right)ds-\;\int_{0}^{\eta }\int_{0}^{t}\phi_{q}\left(\frac{1}{w\left(s\right)}\right)w_{q}\left(\int_{0}^{s}y\left(\tau \right)d\tau \right)ds\;dA\left(t\right)\right].$$

where

(2.20) $$\begin{array}{l} \rho \left(t\right)=re^{-t},\;\\ \;\;\;\;\;r=\frac{1}{\int_{0}^{\infty }\phi_{q}(\frac{1}{w\left(s\right)})\phi_{q}(\int_{0}^{s}y(\tau )d\tau )ds-\int_{0}^{\eta }\int_{0}^{t}\phi_{q}(\frac{1}{w\left(s\right)})\phi_{q}(\int_{0}^{s}y(\tau )d\tau )dsdA(t)}, \end{array}$$

and

$$\int_{0}^{\infty }\phi_{q}\left(\frac{1}{w\left(s\right)}\right)\phi_{q}\left(\int_{0}^{s}y\left(\tau \right)d\tau \right)ds\;-\;\int_{0}^{\eta }\int_{0}^{t}\phi_{q}\left(\frac{1}{w\left(s\right)}\right)\omega_{q}\left(\int_{0}^{s}y\left(\tau \right)d\tau \right)ds\;dA\left(t\right)\neq 0.$$

Then

$$Q^{2}y=Q\left(Qy\right)=r\times \frac{1}{r}\left(Qy\right)=Qy,\;y\in \;Z,$$

and Q(λy)=λQy, y∈Z, λ∈R.

Therefore Q is a semi-projector with

 dimkerL=dim ImQ=1.

We define R(x,λ): X×[0, 1]→X by

(2.21) $$\begin{array}{ll} R\left(x,\lambda \right) & =\frac{1}{\left(n-2\right)!}\int_{0}^{t}{\left(t-s\right)}^{n-2}\phi_{q}\left(\frac{1}{w\left(s\right)}\right)\phi_{q}\left(\int_{0}^{s}\left(I-Q\right)N_{\lambda }x\left(\tau \right)d\tau \right)ds\\ & {-\;\frac{1}{\left(n-2\right)!}\int_{0}^{\eta }(\eta -\;)}^{n-2}\phi_{q}\left(\frac{1}{w\left(s\right)}\right)\phi_{q}\left(\int_{0}^{s}\left(I-Q\right)N_{\lambda }x\left(\tau \right)d\tau \right)ds \end{array}$$

(2.1), 2.2) and (2.3) are easily verified. We verify (2.4) and (2.5).

Let P:X→kerL be defined by

(2.22) $$Px\left(t\right)=\frac{x^{\left(n-2\right)}\left(0\right)t^{n-2}}{\left(n-2\right)!},t\;\in \left(0,\infty \right).$$

For $x\;\in \Sigma_{\lambda }=\left\{x\;\in \Omega :\;Lx=\;N_{\lambda }x\right\}$

(2.23) $$\left(w\left(t\right)\phi_{p}\left(x^{\left(n-1\right)}\left(t\right)\right)\right)'=\lambda h\left(t,\;x\left(t\right),\;x'\left(t\right),\cdots ,\;x^{\left(n-1\right)}\left(t\right)\right).$$

Therefore

$$\begin{array}{ll} R\left(x,\lambda \right)\left(t\right) & =\frac{1}{\left(n-2\right)!}\int_{0}^{t}{\left(t-s\right)}^{n-2}\phi_{q}\left(\frac{1}{w\left(s\right)}\right)\phi_{q}\left(\int_{0}^{s}\left(I-Q\right)N_{\lambda }\;x\left(\tau \right)d\tau \right)ds\\ & -\;\frac{1}{\left(n-2\right)!}\;\int_{0}^{\eta }{\left(\eta -s\right)}^{n-2}\phi_{q}\left(\frac{1}{w\left(s\right)}\right)\phi_{q}\left(\int_{0}^{s}\left(I-Q\right)N_{\lambda }x\left(\tau \right)d\tau \right)ds\\ & =\frac{1}{\left(n-2\right)!}\int_{0}^{t}{\left(t-s\right)}^{n-2}\phi_{q}\left(\frac{1}{w\left(s\right)}\right)\phi_{q}\left(\int_{0}^{s}\left(\text{ w}\left(\tau \right)\phi_{p}\left(\;x^{\left(n-1\right)}\left(\tau \right)\right)\right)'d\tau \right)ds\\ & -\frac{1}{\left(n-2\right)!}\int_{0}^{\eta }{\left(\eta -s\right)}^{n-2}\phi_{q}\left(\frac{1}{w\left(s\right)}\right)\phi_{q}\left(\int_{0}^{s}\left(w\left(\tau \right)\phi_{p}\left(\;x^{\left(n-1\right)}\left(\tau \right)\right)\right)'d\tau \right)ds\\ & =\frac{1}{\left(n-2\right)!}\;\int_{0}^{t}{\left(t-\;s\right)}^{n-2}x^{\left(n-1\right)}\left(s\right)ds\;-\frac{1}{\left(n-2\right)!}\;\int_{0}^{\eta }{\left(\eta -\;s\right)}^{n-2}x^{\left(n-1\right)}\left(s\right)ds\\ & \;=x\left(t\right)\;-\;\left(x\left(0\right)\;+\;x'\left(0\right)t\;+\cdots +\frac{x\left(n-2\right)\left(0\right)}{\left(n-2\right)!}t^{n-2}\right)\;-\;x\left(\eta \right)\;+\;x\left(0\right)\;\\ & +\;x'\left(0\right)\eta \;+\cdots +\frac{x^{\left(n-2\right)}\left(0\right)}{\left(n-2\right)!}\eta^{n-2} \end{array}$$

From the boundary conditions $x^{\left(n-1\right)}\left(0\right)=x^{\left(i\right)}\left(0\right)=0,\;i=1,\;2,\cdots ,\;n-3$ we obtain

$$R\left(x,\lambda \right)\left(t\right)\;=\;x\left(t\right)-\;\frac{x^{\left(n-2\right)}\left(0\right)}{\left(n-2\right)!}t^{n-2}\;-\;x\left(\eta \right)\;+\;\frac{x^{\left(n-2\right)}\left(0\right)}{\left(n-2\right)!}\eta^{n-2}$$

and from $x^{\left(n-2\right)}\left(0\right)=\frac{\left(n-2\right)!}{\eta^{n-2}}x\left(\eta \right)$ we derive

(2.24) $$R\left(x,\;\lambda \right)\left(t\right)\;=\;x\left(t\right)-\frac{x^{\left(n-2\right)}\left(0\right)}{\left(n-2\right)!}\;t^{n-2}=\left(I-\;P\right)x\left(t\right).$$

Also

(2.25) LP + Rx,λt=wtϕpxn−20n−2!tn−2 + 1n−2!∫0tt −sn−2ϕq1wsϕq∫0sI−QNλxτdτds− 1n−2!∫0η η−sn−2ϕq1wsϕq∫0sNλI−QNλxτdτ dsn−1'=wtϕpϕq1wsϕq∫0tI−QNλ xτdτ'=I−QNλ xt

This verifies (2.4) and (2.5). Next, we show that R(x,λ) is compact. Let  r= sup {||x||:x∈Ω}. Since $h:\;\left[0,\;\infty \right)\times {\mathbb{R}}^{n}\to \mathbb{R}$ is $L^{1}$ Caratheodory there exists $\psi_{r}\in L^{1}\left[0,\infty \right)$ such that for x ∈Ω and a. e. t∈[0, ∞)

(2.26) $$\;\left|h\left(t,\;x\left(t\right),\;x'\left(t\right),\cdots ,\;x^{\left(n-1\right)}\left(t\right)\right)\right|\leq \psi_{r}\left(t\right).$$

$$\begin{array}{ll} \left|QN_{\lambda }\;x\right| & \leq \;re^{-t}\;[\int_{0}^{\infty }\phi_{q}\left(\frac{1}{\omega (s)}\right)\phi_{q}\left(\int_{0}^{s}\lambda |h\left(\tau ,\;x\left(\tau \right),\;x'\left(\tau \right),\;\cdots ,\;x^{\left(n-1\right)}\left(\tau \right)\right)d\tau |\right)ds\;\\ & +\int_{0}^{\eta }\int_{0}^{t}\phi_{q}\left(\frac{1}{\omega (s)}\right)\phi_{q}\left(\int_{0}^{s}\;\lambda |h\left(\tau ,\;x\left(\tau \right),\;x'\left(\tau \right),\cdots ,\;x^{\left(n-1\right)}\left(\tau \right)\right)d\tau |\right)ds\;dA\left(t\right)]\\ & \leq re^{-t}\left[||\phi_{q}\left(\frac{1}{w}\right)|{|}_{1}\phi_{q}\left(||\phi_{r}|{|}_{1}\right)\;+\;{\left|\left|\phi_{q}\left(\frac{1}{w}\right)\right|\right|}_{1}\phi_{q}\left({\left|\left|\psi_{r}\right|\right|}_{1}\right)\;\left(A\left(\eta \right)\;-\;A\left(0\right)\right)\right]\\ & \leq \left(r\;+\;1\right){\left|\left|\phi_{q}\left(\frac{1}{w}\right)\right|\right|}_{1}\phi_{q}\left({\left|\left|\psi_{r}\right|\right|}_{1}\right). \end{array}$$

Thus

(2.27) $${\left||QN_{\lambda }|\right|}_{1}\leq \left(r\;+\;1\right){\left||\phi_{q}\left(\frac{1}{w}\right)|\right|}_{1}\phi_{q}\left({\left||\psi_{r}|\right|}_{1}\right).$$

For  x∈Ω  and setting

(2.28) $$A_{n}=\;\max_{0\leq i\leq n-2}\left(sup_{t\in [0,\infty )}\;e^{-t}t^{n-2-i}\right).$$

$$\begin{array}{l} \text{We}\;\text{have}\;e^{-t}\left|R\left(x,\lambda \right)\left(t\right)\right|\leq \;e^{-t}\left[\begin{array}{l} \int_{0}^{t}\;{\left(t-\;s\right)}^{n-2}\phi_{q}\left(\frac{1}{\omega }\right)\times \\ \phi_{q}\left(\int_{0}^{s}\left|\left(I\;-\;Q\right)\lambda h\left(\tau ,\;x\left(\tau \right),\;x'\left(\tau \right),\cdots ,\;x^{\left(n-1\right)}\left(\tau \right)\right)d\tau \right|\right)ds \end{array}\right]\\ \;\;\;\;\;\;\;\;\;\;\;\;\;\;\;\;\;\;\;\;\;\;\;\;\;\;\;\;\;\;\;\;\;\;+\;\int_{0}^{\eta }{\left(\eta \;-\;s\right)}^{n-2}\phi_{q}\left(\frac{1}{\omega (s)}\right)\phi_{q}\left(\int_{0}^{s}\left|\left(I\;-\;Q\right)\lambda \;h\left(\tau ,\;x\left(\tau \right),\;x'\left(\tau \right),\;\cdots ,\;x^{n-1}\left(\tau \right)\right)d\tau \right|\right)ds]\\ \;\;\;\;\;\;\;\;\;\;\;\;\;\;\;\;\;\;\;\;\;\;\;\;\;\;\;\;\;\;\;\;\;\;\leq Sup_{t\in [0,\infty )}e^{-t}t^{n-2}{\left|\left|\phi_{q}\left(\frac{1}{\omega }\right)\right|\right|}_{1}\phi_{q}\left({\left|\left|\;\psi_{r}\right|\right|}_{1}+\;{\left|\left|\;Q\;N_{\lambda }\right|\right|}_{1}\right)\\ \;\;\;\;\;\;\;\;\;\;\;\;\;\;\;\;\;\;\;\;\;\;\;\;\;\;\;\;\;\;\;\;\;\;+\;sup_{t\in [0,\infty )}e^{-t}t^{n-2}{\left|\left|\phi_{q}\left(\frac{1}{\omega }\right)\right|\right|}_{1}\phi_{q}\left({\left|\left|\psi_{r}\right|\right|}_{1}+\;{\left|\left|\;Q\;N_{\lambda }\right|\right|}_{1}\right)\\ \;\;\;\;\;\;\;\;\;\;\;\;\;\;\;\;\;\;\;\;\;\;\;\;\;\;\;\;\;\;\;\;\;\;=\;2\;sup_{t\in [0,\infty )}e^{-t}t^{n-2}\left[\;\left({\left|\left|\phi_{q}\left(\frac{1}{\omega }\right)\right|\right|}_{1}\right)\phi_{q}\left({\left|\left|\psi_{r}\right|\right|}_{1}+\;{\left|\left|\;Q\;N_{\lambda }\right|\right|}_{1}\right)\right]\leq 2A_{n}\left[\left({\left|\left|\phi_{q}\left(\frac{1}{\omega }\right)\right|\right|}_{1}\right)\phi_{q}\left({\left|\left|\psi_{r}\right|\right|}_{1}+\;{\left|\left|\;Q\;N_{\lambda }\right|\right|}_{1}\right)\right]. \end{array}$$

For 1 ≤i ≤n−2 we have

$$\begin{array}{ll} e^{-t}\left|R{\left(x,\;\lambda \right)}^{\left(i\right)}\left(t\right)\right| & =e^{-t}\left|\frac{1}{\left(n-2-i\right)}\int_{0}^{t}{\left(t-s\right)}^{n-2-i}\phi_{q}\left(\frac{1}{\omega (s)}\right)\phi_{q}\left(\int_{0}^{s}\left(I-Q\right)N_{\lambda }x\left(\tau \right)d\tau \right)ds\right|\\ & \leq \max_{0\leq i\leq n-2}sup_{t\in [0,\infty )}\;e^{-t}t^{n-2-i}{\left||\phi_{q}\left(\frac{1}{\omega }\right)|\right|}_{1}\phi_{q}\left({\left||\psi_{r}|\right|}_{1}+\;{\left||\;Q\;N_{\lambda }|\right|}_{1}\right)\\ & \leq A_{n}{\left||\phi_{q}\left(\frac{1}{\omega }\right)|\right|}_{1}\phi_{q}\left({\left||\psi_{r}|\right|}_{1}+\;{\left||\;Q\;N_{\lambda }|\right|}_{1}\right). \end{array}$$

For  i = n−1 we have

$$e^{-t}\left|R{\left(x,\lambda \right)}^{\left(n-1\right)}\left(t\right)\right|\;\leq {\left||\phi_{q}\left(\frac{1}{\omega }\right)|\right|}_{\infty }\phi_{q}\left({\left||\psi_{r}|\right|}_{1}+\;{\left||\;Q\;N_{\lambda }|\right|}_{1}\right).$$

Therefore

(2.29) $$\left|\left|R\left(x,\;\lambda \right)\right|\right|\leq max\left(2A_{n}{\left|\left|\phi_{q}\left(\frac{1}{w}\right)\right|\right|}_{1}\text{, }{\left|\left|\phi_{q}\left(\frac{1}{w}\right)\right|\right|}_{\infty }\right)\phi_{q}\left({\left|\left|\psi_{r}\right|\right|}_{1}+\;{\left|\left|\;Q\;N_{\lambda }\right|\right|}_{1}\right)\;=\;L_{n}.$$

Thus R(. , λ) is uniformly bounded in X.

For any $t_{1},\;t_{2}\in \left[0,\;D\right],\;D\in \left(0,\infty \right),\;t_{1}<\;t_{2}$ we have

$$\begin{array}{ll} \left|e^{-t_{2}}R\left(x,\;\lambda \right)\left(t_{2}\right)\;-\;e^{-t_{1}}\;R\left(x,\lambda \right)\left(t_{1}\right)\;\right| & =\left|\int_{t_{1}}^{t_{2}}\left[e^{-\tau }R\left(x,\;\lambda \right)\left(\tau \right)\right]'d\tau \right|\\ & =\left|\int_{t_{1}}^{t_{2}}\left[-e^{-\tau }R'\left(x,\;\lambda \right)\left(\tau \right)\right]'d\tau \;+\;\int_{t_{1}}^{t_{2}}\left[e^{-\tau }R'\left(x,\;\lambda \right)\left(\tau \right)\right]'d\tau \right|\\ & \leq 2\;\left(t_{2}-\;t_{1}\right)\left||R\left(x,\;\lambda \right)|\right|\to \;0\;as\;t_{1}\to \;t_{2}. \end{array}$$

For 1≤i≤n−2 we have

$$\begin{array}{l} \begin{array}{ll} \left|e^{-t_{2}}R^{\left(i\right)}\left(x,\;\lambda \right)\left(t_{2}\right)\;-\;e^{-t_{1}}R^{\left(i\right)}\left(x,\lambda \right)\left(t_{1}\right)\;\right| & =\;\left|\int_{t_{1}}^{t_{2}}\left[e^{-s}R^{\left(i\right)}\left(x,\;\lambda \right)\left(s\right)\right]'ds\right|\\ & =\;\left|\int_{t_{1}}^{t_{2}}\left[-e^{-s}R^{\left(i\right)}\left(x,\;\lambda \right)\left(s\right)\;+\;e^{-s}R^{\left(i+1\right)}\left(x,\;\lambda \right)\left(s\right)\right]\;ds\right|\\ & \;\leq 2\;\left(t_{2}-\;t_{1}\right)\left||R\left(x,\;\lambda \right)|\right|\to \;0\;as\;t_{1}\to t_{2}. \end{array} \end{array}$$

For  i = n−1 we have

$$\begin{array}{ll} |e^{-t_{2}}\phi_{p}R^{\left(n-1\right)}\left(x,\;\lambda \right)\left(t_{2}\right)-\;e^{-t_{1}}\phi_{p}\;R^{\left(n-1\right)}\left(x,\;\lambda \right)\left(t_{1}\right)\\ & =\left|\frac{e^{-t_{2}}}{\omega (t_{2})}\int_{0}^{t_{2}}\;\left(I-Q\right)N_{\lambda }x\left(\tau \right)d\tau -\frac{e^{-t_{1}}}{\omega (t_{1})}\int_{0}^{t_{1}}\;\left(I-Q\right)N_{\lambda }x\left(\tau \right)d\tau \right|\\ & \leq \left|\;\frac{e^{-t_{2}}}{\omega (t_{2})}\;-\frac{e^{-t_{1}}}{\omega (t_{1})}\right|\int_{0}^{t_{2}}\left|\left(I-Q\right)N_{\lambda }x\left(\tau \right)\right|d\tau \;+\frac{e^{-t_{1}}}{\omega (t_{1})}\;\left|\int_{t_{1}}^{t_{2}}\left|\left(I-Q\right)N_{\lambda }x\left(\tau \right)\right|d\tau \right|\\ & \;\leq {\left|\left|\frac{1}{\omega }\right|\right|}_{\infty }^{2}\left|\omega \left(t_{1}\right)e^{-t_{2}}-\;\omega \left(t_{2}\right)e^{-t_{1}}\right|\int_{0}^{t_{2}}\left(\left|\psi_{r}\left(s\right)\right|\;+\;|QN_{\lambda }\left(s\right)|\right)ds\\ & +\;{\left|\left|\frac{1}{\omega }\right|\right|}_{\infty }\int_{t_{1}}^{t_{2}}\left(\left|\psi_{r}\left(s\right)\right|\;+\;|QN_{\lambda }\left(s\right)|\right)ds\\ & \leq {\left|\left|\frac{1}{\omega }\right|\right|}_{\infty }^{2}|\omega \left(t_{1}\right)e^{-t_{2}}-\omega \left(t_{2}\right)e^{-t_{1}}|(||\psi_{r}|{|}_{1}+||QN_{\lambda }|{|}_{1})\\ & +\;{\left|\left|\frac{1}{\omega }\right|\right|}_{\infty }\int_{t_{1}}^{t_{2}}\left(\left|\psi_{r}\left(s\right)\right|\;+\;|QN_{\lambda }\left(s\right)|\right)ds\to \;0\;as\;t_{1}\to t_{2}|. \end{array}$$

This shows that

$$\left|e^{-t_{2}}R^{\left(n-1\right)}\left(x,\lambda \right)\left(t_{2}\right)\;-\;e^{-t_{1}}\;R^{\left(n-1\right)}\left(x,\;\lambda \right)\left(t_{1}\right)\;\right|\;\to 0\;as\;t_{1}\to t_{2}.$$

Thus R(x, λ) is equicontinuous on every compact subset of [0, ∞). Next we show that $R\left(x,\;\lambda \right)\left(\bar{\Omega }\right)$ is equiconvergent at infinity. We have

$$e^{-t}\left|\;R\left(x,\;\lambda \right)\left(t\right)\right|\leq 2e^{-t}t^{n-1}[\phi_{q}\left({\left|\left|\psi_{r}\right|\right|}_{1}+\;\left|QN_{\lambda }\right|{|}_{1}\right)\left({\left|\left|\;\phi_{q}(\frac{1}{\omega })\right|\right|}_{1}\right]\;\to 0\;as\;t\;\to \infty .$$

For 1≤i≤n−2 we have

$$e^{-t}\left|\;R^{\left(i\right)}\left(x,\;\lambda \right)\left(t\right)\right|\leq e^{-t}t^{n-2-i}{\left|\left|\;\phi_{q}(\frac{1}{\omega })\right|\right|}_{1}\phi_{q}\left({\left|\left|\psi_{r}\right|\right|}_{1}\;+\;|\left|QN_{\lambda }\right|{|}_{1}\right)\;\to 0\;as\;t\;\to \infty .$$

For i = n− 1  we have

$$e^{-t}\left|\;R^{\left(n-1\right)}\left(x,\lambda \right)\left(t\right)\right|\leq e^{-t}{\left||\;\phi_{q}\left(\frac{1}{\omega }\right)|\right|}_{\infty }\phi_{q}\left[{\left||\psi_{r}|\right|}_{1}+\;{\left||QN_{\lambda }|\right|}_{1}\right]\;\to 0\;as\;t\;\to \infty .$$

Thus R(x, λ)(ϖ) is equiconvergent at infinity. Hence from the above computations R(x, λ) is compact. The continuity of R(x, λ) follows from the Lebesgue dominated convergence theorem. Thus $N_{\lambda }$ is L-compact in ϖ.

## 3 Existence result

In what follows we shall assume the following conditions

$\;\left(H_{1}\right)$ There exist positive functions $a_{i}\in L^{1}\left[0,\;\infty \right)\;i=\;0,\;1,\;2,\cdots ,\;n-1\;$ with

(3.1) $$2^{q-2}A_{n}{\left||\phi_{q}\left(\frac{1}{\omega }\right)|\right|}_{1}{\left(\sum_{i=1}^{n-1}{\left||a_{i}|\right|}_{1}\right)}^{q-1}<\;1\;if\;p\leq 2,$$

or

(3.2) $$A_{n}{\left||\phi_{q}\left(\frac{1}{\omega }\right)|\right|}_{1}{\left(\sum_{i=1}^{n-1}{\left||a_{i}|\right|}_{1}\right)}^{q-1}<\;1\;if\;p\;>\;2,$$

such that

(3.3) $$\left|h(t,\;x_{1},\;x_{2},\;\cdots ,\;x_{n}\right|\;\leq a_{0}\left(t\right)\;+\;\sum_{i=1}^{n}a_{i}\phi_{p}\left(e^{-t}\left|x_{i}\right|\right),\;x\in \mathbb{R}.$$

$\left(H_{2}\right)$ There exists a constant $M_{n}>0$ such that for $\left|x^{\left(n-2\right)}\left(t\right)\right|>\;M_{n}$, for all t ∈ [0, ∞) we have $QN_{\lambda }x\;\neq 0$.

$\left(H_{3}\right)$ $r\;=\int_{0}^{\infty }\phi_{q}\left(\frac{1}{w\left(s\right)}\right)\phi_{q}\left(\int_{0}^{s}e^{-\tau }d\tau \right)ds\;-\;\int_{0}^{\eta }\int_{0}^{t}\phi_{q}\left(\frac{1}{w\left(s\right)}\right)\phi_{q}\left(\int_{0}^{s}e^{-\tau }d\tau \right)ds\;dA\left(t\right)\neq 0$.

$\left(H_{4}\right)$ There exists constant $M_{n}^{*}>0$ such that for

$x\left(t\right)=\;ct^{n-2}\in kerL\;with\;\left|c\right|>\frac{M_{n}^{*}}{\left(n-2\right)!}$ we have either

(3.4) $$cQN_{\lambda }x\;>\;0$$

or

(3.5) $$cQN_{\lambda }x\;<\;0.$$

**Theorem 3.1:** If $\;\left(H_{1}\right)$- $\left(H_{4}\right)$ is satisfied then the boundary value problem 1.1), 1.2) has at least one solution.

**Proof:** Our objective here is to construct an open bounded set V⊂X that satisfies the assumptions of theorem 2.1. Let

(3.6) $$V_{1}=\left\{x\in domL\;:\;Lx\;=\;N_{\lambda }x,\;\lambda \in \left(0,\;1\right]\right\}.$$

For $x\in V_{1},\;x\notin ker\;L$. Hence $N_{\lambda }x\in Im\;L\;=\;ker\;Q$. Therefore $Q\;N_{\lambda }x\;=0$ and by $\left(H_{2}\right)$ there exists $t_{0}\in \left[0,\infty \right)$ such that

(3.7) $$\left|x^{\left(n-2\right)}\left(t_{0}\right)\right|\leq M_{n}.$$

(3.8) $$\begin{array}{l} \left|x^{\left(n-2\right)}\left(0\right)\right|=\;\left|x^{\left(n-2\right)}\left(t_{0}\right)\;-\int_{0}^{t_{0}}x^{\left(n-1\right)}\left(s\right)ds\;\right|\\ \;\leq {\text{M}}_{n}+|\left|x^{\left(n-1\right)}\right|{|}_{1}. \end{array}$$

For $x\in V_{1},\;\left(I-\;P\right)\;x\in dom\;L\cap \;kerP$. Therefore from (2.24) and (2.29) we obtain

(3.9) $$\left||\left(I-\;P\right)\;x|\right|\;=\;\left||R\left(x,\lambda \right)|\right|\leq \;L_{n}.$$

From the definition of the projection P we derive

(3.10) $${\left(Px\right)}^{\left(i\right)}\left(t\right)=\frac{x^{\left(n-2\right)}\left(0\right)}{\left(n-2-i\right)!}t^{n-2-i},\;i=\;0,\;1,\;2,\cdots ,\;n-2,\;t\in \left(0,\infty \right).$$

(3.11) $$\begin{array}{l} \begin{array}{ll} \left||Px|\right| & =\max_{0\leq i\leq n-2}\frac{x^{\left(n-1\right)}(0)}{(n-2-i)!}sup_{t\in [0,\infty )}e^{-t}t^{n-2-i}\\ & \leq \left|x^{\left(n-2\right)}\left(0\right)\right|\max_{0\leq i\leq n-2}sup_{t\in [0,\infty )}e^{-t}t^{n-2-i}\\ & \leq A_{n}\left|x^{\left(n-2\right)}\left(0\right)\right|\\ & \leq A_{n}\left[M_{n}+\;{\left||x^{\left(n-1\right)}|\right|}_{1}\right]\\ & =\;A_{n}{\left||x^{\left(n-1\right)}|\right|}_{1}+\;A_{n}M_{n}. \end{array} \end{array}$$

(3.12) $$\begin{array}{l} \left||x|\right|\;=\;\left||\;Px\;+\;\left(I\;-\;P\right)\;x|\right|\leq \left||Px|\right|\;+\;\left||\left(I\;-P\right)x\;|\right|\\ \;\;\;\;\;\;\;\leq A_{n}{\left||x^{\left(n-1\right)}|\right|}_{1}+\;A_{n}M_{n}\;+\;L_{n}=\;A_{n}{\left||x^{\left(n-1\right)}|\right|}_{1}+\;B_{n}, \end{array}$$

where $\;B_{n}=\;A_{n}M_{n}+\;L_{n}$.

If p≤2 then from 2.6), 2.16) and (3.3) we obtain

$$\begin{array}{ll} {\left||x^{\left(n-1\right)}|\right|}_{1} & =\int_{0}^{\infty }\left|\phi_{q}\left(\frac{1}{\omega (s)}\right)\phi_{q}(\int_{0}^{s}\lambda h\left(\tau ,\;x\left(\tau \right),\;x'\left(\tau \right),\;\cdots ,\;x^{\left(n-1\right)}\right]\left(\tau \right)d\tau \right|ds\\ & \;\leq {\left||\phi_{q}\left(\frac{1}{\omega }\right)|\right|}_{1}\phi_{q}\left[{\left||a_{0}|\right|}_{1}+\sum_{i=1}^{n}{\left||a_{i}|\right|}_{1}\phi_{p}\left(\left|\right|x\left|\right|\right)\right]\\ & \;\leq {\left||\phi_{q}\left(\frac{1}{\omega }\right)|\right|}_{1}2^{q-2}\left[{\left||a_{0}|\right|}_{1}^{q-1}+\;{\left(\sum_{i=1}^{n}{\left||a_{i}|\right|}_{1}\right)}^{q-1}\left(\left|\right|x\left|\right|\right)\right]. \end{array}$$

From (3.12) we get

$${\left||x^{\left(n-1\right)}|\right|}_{1}\leq {\left||\phi_{q}\left(\frac{1}{\omega }\right)|\right|}_{1}2^{q-2}\left[{\left||a_{0}|\right|}_{1}^{q-1}+\;{\left(\sum_{i=1}^{n}{\left||a_{i}|\right|}_{1}\right)}^{q-1}\left(A_{n}{\left||x^{\left(n-1\right)}|\right|}_{1}+B_{n}\right)\right]$$

or

$$\left(1\;-\;2^{q-2}{\left||\phi_{q}\left(\frac{1}{\omega }\right)|\right|}_{1}{\left(\sum_{i=1}^{n}{\left||a_{i}|\right|}_{1}\right)}^{q-1}A_{n}\right){\left||x^{\left(n-1\right)}|\right|}_{1}\;\leq {\left||\phi_{q}\left(\frac{1}{\omega }\right)|\right|}_{1}2^{q-2}\left[{\left||a_{0}|\right|}_{1}^{q-1}+\;B_{n}{\left(\sum_{i=1}^{n}{\left||a_{i}|\right|}_{1}\right)}^{q-1}\right]\text{ .}$$

(3.13) $${\left|\left|x^{\left(n-1\right)}\right|\right|}_{1}\leq \frac{2^{q-2}||\phi_{q}(\frac{1}{\phi })|{|}_{1}[||a_{0}|{|}^{q-1}+B_{n}(\sum_{i=1}^{n}{\left||a_{i}|{|}_{1}\right)}^{q-1}]}{1-2^{q-2}||\phi_{q}(\frac{1}{\omega })|{|}_{1}((\sum_{i=1}^{n}{\left||a_{i}|{|}_{1}\right)}^{q-1})A_{n}}.$$

Therefore using (3(3.12) and (3.1)(3.1) we conclude that there exists $D_{n}>0$ such that

(3.14) $$\left||x|\right|<\;D_{n}.$$

Similarly if p>2

(3.15) $${\left|\left|x^{\left(n-1\right)}\right|\right|}_{1}\leq \frac{||\phi_{q}(\frac{1}{\omega })|{|}_{1}[||a_{0}|{|}^{q-1}+B_{n}(\sum_{i=1}^{n}{\left||a_{i}|{|}_{1}\right)}^{q-1}]}{1-||\phi_{q}(\frac{1}{\omega })|{|}_{1}(\sum_{i=1}^{n}{\left||a_{i}|{|}_{1}\right)}^{q-1}A_{n}}.$$

Again from (3(3.12) and (3.2)(3.2) we obtain $\left||x|\right|\;<\;D_{n}^{*}$ for some $D_{n}^{*}>\;0$.

Therefore $V_{1}$ is bounded.

Let $V_{2}=\left\{x\in kerL\;:\;N_{\lambda }x\;\in \;ImL\right\}$.

For $x\in V_{2},\;x\left(t\right)\;=\;ct^{n-2},\;c\in R,\;t\;\in \;\left(0,\;\infty \right)$

(3.16) $$N_{\lambda }x\in \;ImL\;implies\;N_{\lambda }\;x\;\in ker\;Q\;and\;hence\;QN_{\lambda }x\;=0.$$

From $\left(H_{4}\right)$ we obtain

(3.17) $$\left|c\right|<\;\frac{M_{n}^{*}}{\left(n-2\right)!}.$$

Therefore for $x\in V_{2}$,

(3.18) $$\left|\left|x\right|\right|=\;\left|c\right|\max_{0\leq i\leq n-2}(sup_{t\in [0,\infty )}e^{-t}\left(t^{\left(n-2)(i\right)}\right)\leq M_{n}^{*}A_{n}.$$

Thus $V_{2}$ is bounded.

We choose $M_{0}$ large enough such that

$$V=\left\{x\in V:\;\left|\left|x\right|\right|\;<\;M_{0}\right\}⊃\bar{V_{1}}\;\cup \bar{V_{2}}$$

Therefore from the above computations

$$Lx\neq N_{\lambda }x\;for\;x\in \partial V\cap domL.$$

Thus the first part of theorem 2.1 is verified.

(3.19) LetH(x,λ)=λx + (1−λ) JQN x, λ∈[0, 1].

Where J: ImQ→kerL is the homeomorphism defined by

(3.20) $$J\left(\rho c\right)\;=\;ct^{n-2}.$$

Then for $\;x\in \partial V\;\cap kerL,\;x\;=\;ct^{n-2}\neq 0$$H\left(x,\;1\right)\;=\lambda c\;t^{n-2}\neq 0$ and H(x, 0)= JQNx≠0, since Nx∉ Im L.

Therefore for λ=0 or λ= 1 ,H(x,λ)≠0. Let 0 <λ<1. Suppose H(x,λ) = 0  then from (3.19) and (3.4) we have

$$\;-c^{2}=\;c\frac{\left(1-\lambda \right)}{\lambda }QN\left(ct^{n-2}\right)>0,$$

which is a contradiction. Similarly using

H(x,λ) =− λx + (1−λ) JQNx = 0 and (3.5) we obtain the contradiction

$$c^{2}=\;\frac{1-\lambda }{\lambda }\;c\;QN\left(ct^{n-2}\right)\;<0.$$

Thus H(x, λ)≠0 for x ∈∂ V∩kerL, λ∈ [0, 1]. Therefore by the invariance of the degree under a homotopy we derive

deg(JQN, V∩ker L, 0) = deg (H(., 1), V∩kerL, 0)

= deg (H(., 0), V∩kerL, 0)

=deg (±1, V∩kerL, 0)≠0.

Therefore we conclude from theorem 2.1 that 1.1), 1.2) has at least one solution.

## 4 Example

We consider the following boundary value problem

$$(w\left(t\right)\phi_{p}\left(x'''\left(t\right)\right))'=\;3\;+\;e^{-t\left(p-1\right)}[\frac{|x\left(t\right){|}^{2}}{4{\left(1+t\right)}^{2}}+\;\frac{|x'\left(t\right){|}^{2}}{8{\left(1+t\right)}^{3}}$$

(4.1) $$+\;\frac{|x''(t){|}^{2}}{{16(1+t)}^{4}}+\;\left|\;cos\;t\right|{\left|x'''\left(t\right)\right|}^{2}].$$

(4.2) $$\;x''\left(0\right)=2x\left(1\right),\;x'''\left(0\right)=x'\left(0\right)=x\left(0\right)=0,\;x''\left(\infty \right)=\int_{0}^{1}x''\left(s\right)ds\text{ .}$$

Here

$$\omega \left(t\right)=e^{t},\;t\;\in \left[0,\infty \right),\;p=\frac{3}{2},\;q=3,\;\eta =1,\;A\left(s\right)=s,\;n=4.$$

$$h\left(t,\;x,\;x',\;x'',\;x'''\right)=\;3\;+\;e^{-\frac{t}{2}}\left[\frac{|x\left(t\right){|}^{2}}{4{\left(1+t\right)}^{2}}+\;\frac{|x'\left(t\right){|}^{2}}{8{\left(1+t\right)}^{3}}+\;\frac{|x''\left(t\right){|}^{2}}{16{\left(1+t\right)}^{4}}+\left|\cos\;t\right|\;{\left|x'''\left(t\right)\right|}^{2}\right].$$

$$a_{1}\left(t\right)=\frac{1}{{4\left(1+t\right)}^{2}},\;a_{2}\left(t\right)=\frac{1}{{8\left(1+t\right)}^{3}},\;a_{3}\left(t\right)=\frac{1}{{16\left(1+t\right)}^{4}}.$$

$${\left||a_{1}|\right|}_{1}=\frac{1}{4},\;{\left||a_{2}|\right|}_{1}=\frac{1}{16},\;{\left||a_{3}|\right|}_{1}=\frac{1}{48}.$$

$$\begin{array}{l} A_{n}=\max_{0\leq i\leq n-1}(sup_{t\in [0,\infty )}e^{-t}t^{n-2-i})\\ \;=\max_{0\leq i\leq n-1}(sup_{t\in [0,\infty )}e^{-t}t^{2-i}\;\\ \;=max_{t\in [0,\infty )}\left[t^{2}e^{-t},\;t\;e^{-t},\;e^{-t}\right]=1. \end{array}$$

$$\sum_{i=1}^{n-1}{\left||a_{i}|\right|}_{1}=\sum_{i=1}^{3}{\left||a_{i}|\right|}_{1}=\left(\frac{1}{4}+\;\frac{1}{16}\;+\frac{1}{48}\right)=\frac{1}{3}.$$

$${\left||\phi_{q}\left(\frac{1}{\omega }\right)|\right|}_{1}=\int_{0}^{\infty }e^{-2t}dt\;=\frac{1}{2}.$$

Thus,

$$2^{q-2}A_{n}{\left||\phi_{q}\left(\frac{1}{\omega }\right)|\right|}_{1}{\left(\sum_{i=1}^{n-1}{\left||a_{i}|\right|}_{1}\right)}^{q-1}=\;2\;A_{n}{\left||\phi_{q}\left(\frac{1}{\omega }\right)|\right|}_{1}{\left(\sum_{i=1}^{3}{\left||a_{i}|\right|}_{1}\right)}^{2}=2\;\times 1\times \frac{1}{2}\times {\left(\frac{1}{3}\right)}^{2}=\frac{1}{9}<1.$$

and

$$\left|h(t,\;x,\;x',\;x'',\;x'''\right|\leq 3+\;e^{-\frac{t}{2}}\left[\frac{\left|x(t)\right|}{{4(1+t)}^{2}}+\frac{\left|x'(t)\right|}{{8(1+t)}^{3}}\;+\;\frac{\left|x''(t)\right|}{{16(1+t)}^{4}}+\;\left|x'''\left(t\right)\right|\right].$$

This verifies $H_{1}$.

$$\begin{array}{l} r=\int_{0}^{\infty }w_{q}\left(\frac{1}{w\left(s\right)}\right)\phi_{q}\left(\int_{0}^{s}e^{-\tau }d\tau \;\right)ds\;-\int_{0}^{\eta }\int_{0}^{t}\phi_{q}\left(\frac{1}{w\left(s\right)}\right)\phi_{q}\left(\int_{0}^{s}e^{-\tau }d\tau \;\right)dsdt\\ \;\;\;=\int_{0}^{1}\int_{0}^{\infty }\phi_{q}\left(\frac{1}{w\left(s\right)}\right)\phi_{q}\left(\int_{0}^{s}e^{-\tau }d\tau \right)dsdt\;-\int_{0}^{1}\int_{0}^{t}\phi_{q}\left(\frac{1}{w\left(s\right)}\right)\phi_{q}\left(\int_{0}^{s}e^{-\tau }d\tau \;\right)dsdt\\ \;\;\;=\int_{0}^{1}\int_{t}^{\infty }\phi_{q}\left(\frac{1}{w\left(s\right)}\right)\phi_{q}\left(\int_{0}^{s}e^{-\tau }d\tau \right)dsdt\;>0. \end{array}$$

$$\begin{array}{l} QN_{\lambda }x=\rho [\int_{0}^{\infty }\phi_{q}\left(\frac{1}{w\left(s\right)}\right)\phi_{q}\left(\int_{0}^{s}N_{\lambda }\left(\tau \right)d\tau \;\right)ds\\ -\;\int_{0}^{1}\int_{0}^{t}\phi_{q}\left(\frac{1}{w\left(s\right)}\right)\phi_{q}\left(\int_{0}^{s}N_{\lambda }\left(\tau \right)d\tau \right)dsdt\\ \;=\rho \int_{0}^{1}\int_{t}^{\infty }\phi_{q}\left(\frac{1}{w\left(s\right)}\right)\phi_{q}\left(\int_{0}^{s}N_{\lambda }\left(\tau \right)d\tau \;\right)dsdt\; \end{array}$$

Since h(t, x, y, z,ω)>0 for all $\left(t,\;x,\;y,\;z,\;\omega \right)\in \left[0,\;\infty \right)\times {\mathbb{R}}^{4}$ and $\rho \left(t\right)\;=\;re^{-t}>0$ then $QN_{\lambda }x\left(t\right)\neq 0$ on [0, ∞). This verifies $H_{2}$ and $H_{3}$.To verify $H_{4}$,

Let $x\left(t\right)\;=\;ct^{n-2}=\;c\;t^{2}\in ker\;L$. Then,

$$c\;QN_{\lambda }x\left(t\right)\;=\;c\int_{0}^{1}\int_{t}^{\infty }e^{-2s}[{\int_{0}^{s}\left(3\;+\;e^{-\frac{\tau }{2}}\left(\frac{|c\tau {|}^{2}}{{4(1+\tau )}^{2}}+\;\frac{|2c\tau {|}^{2}}{{8(1+\tau )}^{3}}+\;\frac{|2c\tau {|}^{2}}{{16(1+\tau )}^{4}}\right)d\tau \;ds\;dt\right]}^{2}\text{ .}$$

If c >1 then,

$$cQN_{\lambda }x\geq c\int_{0}^{1}\int_{t}^{\infty }9\;s^{2}e^{-2s}\;ds\;dt>0,$$

and if c< −1  then,

$$cQN_{\lambda }x\;<\;c\int_{0}^{1}\int_{t}^{\infty }9\;s^{2}e^{-2s}\;ds\;dt\;<0.$$

Therefore assumptions (3.4) or (3.5) are satisfied respectively if

$$\left|c\right|>\frac{M_{n}^{*}}{\left(n-2\right)!}=\frac{2}{2!}=1.$$

Thus all the assumptions of theorem 3.1 are satisfied. Hence example 4.1–4.2 has at least one solution.

## Declarations

### Author contribution statement

S. A. Iyase: Conceived and designed the experiments; Analyzed and interpreted the data; Wrote the paper..

K. S. Eke: Analyzed and interpreted the data.

### Funding statement

This work was supported by 10.13039/501100006335Covenant University.

### Competing interest statement

The authors declare no conflict of interest.

### Additional information

No additional information is available for this paper.
